# Patients’ perspectives of endometriosis-related fatigue: qualitative interviews

**DOI:** 10.1186/s41687-020-00200-1

**Published:** 2020-05-06

**Authors:** Dana DiBenedetti, Ahmed M. Soliman, Catherine Gupta, Eric S. Surrey

**Affiliations:** 1 RTI Health Solutions, 3040 East Cornwallis Road, Post Office Box 12194, Research Triangle Park, NC 27709-2194 USA; 2 AbbVie Inc., North Chicago, IL USA; 3 Colorado Center for Reproductive Medicine, Lone Tree, CO USA

**Keywords:** Endometriosis, Fatigue, Quality of life, Qualitative, Interview

## Abstract

**Background:**

Endometriosis-related fatigue is common and negatively impacts multiple areas of many women’s lives, particularly in day-to-day activities, social activities, physical activities, mood and emotions, relationships with family or partners, and work or school. Multiple studies have documented fatigue as a significant symptom of endometriosis. Additional research is needed to better understand endometriosis-related fatigue and its impacts on patients.

**Methods:**

This qualitative study consisted of individual in-person semistructured interviews conducted with 22 adult females reporting moderate to severe endometriosis-related pain. Women with self-reported, surgically confirmed endometriosis and moderate to severe endometriosis-related pain were recruited from qualitative research firms in two locations in the United States. Qualified subjects participated in semistructured interviews that lasted approximately 45 min each. Interviews were audio recorded and transcribed for qualitative analysis.

**Results:**

All 22 participants reported experiencing fatigue related to their endometriosis. While the degree of severity of their endometriosis-related fatigue varied, 21 of the 22 participants stated that it was at least “somewhat bothersome.” Most participants noted an impact from endometriosis-related fatigue on day-to-day activities, social activities, physical activities, mood and emotions, relationships with family or partner, and work or school.

**Conclusions:**

The data presented here indicate that endometriosis-related fatigue has a pervasive impact on the functioning of women living with this condition. Future studies should measure any changes in fatigue that may be associated with treatment for endometriosis.

## Background

Endometriosis is characterized as an inflammatory condition associated with the menstrual cycle [1]. The prevalence of endometriosis has been estimated to be 6% in the United States (US) [2], although milder cases may be underreported because diagnosis requires surgical confirmation. This condition can have a significant social and psychological impact on the lives of affected women, including negative effects on quality of life, intimate relationships, planning for and having children, education, work, and emotional well-being [3]. Clinical symptoms of endometriosis include severe dysmenorrhea (severe pain or cramping during menses), deep dyspareunia (pain with sexual intercourse), chronic pelvic pain, ovulation-related pain, heavy menstrual bleeding and/or spotting between periods, and painful bowel and/or bladder symptoms that occur during or before menstruation [4]. Although not as often recognized as the aforementioned symptoms, fatigue has been noted as a significant symptom of endometriosis as early as 1995 [5]. Recent studies have reinforced that fatigue is both a common and bothersome symptom among women with endometriosis [6–9]. Endometriosis-related fatigue is commonly accompanied by other symptoms, including menstrual and nonmenstrual pain, anxiety, stress, and irregular bleeding [7–10]. Pain, in particular, has been frequently identified in patients experiencing endometriosis-related fatigue, suggesting that fatigue may be closely related to pain [8, 11].

Although the prevalence of endometriosis-related fatigue has been documented, additional research is needed to characterize patients’ experiences with this symptom, especially in women who experience endometriosis-related pain. Endometriosis-related fatigue can significantly contribute to the impact of this disease on women’s lives, necessitating further investigation into this burdensome symptom to address this gap of qualitative data in the literature. The objective of this study was to better understand the experience of women who suffer from endometriosis-related fatigue as a result of moderate to severe endometriosis-related pain.

## Methods

### Study design

In-depth, in-person, individual interviews were conducted with adult females who reported moderate to severe endometriosis-related pain. The study was granted a review exemption from the RTI International Institutional Review Board, as the research interactions only involved interview procedures. The study followed International Conference on Harmonization Good Clinical Practice guidelines [12]. All interviews were conducted by two of the authors, both of whom are female and have extensive qualitative research experience, including significant experience specific to women’s health. During each interview, one author served as the primary interviewer, and the other took field notes and monitored the need for additional questions or probes. The interviews were designed in accordance with concept elicitation methodology in which concepts relevant to participants’ experience of fatigue symptoms and impacts of fatigue were discussed [13]. Concept saturation (i.e., point at which no new relevant concepts emerge) was also documented, consistent with general recommendations by Willis [13] and the US Food and Drug Administration guidance on patient-reported outcomes [14]. Interviews lasted approximately 45 min each and were audio recorded and transcribed for analysis.

### Data analysis

Immediately following each interview, interviewers began the analysis process through debriefing and recording initial thoughts. Additional analysis was facilitated by field notes and verbatim transcripts to address the study objectives. Specifically, dominant trends were identified in each interview and then compared across the results of the other interviews to generate themes or patterns in the way participants talk about their endometriosis-related fatigue. Data were analyzed using coding software (ATLAS-ti version 7.5; Scientific Software Development GmbH), and the initial coding framework was adapted to incorporate additional codes based on emerging themes. One interviewer coded all transcripts. Participant identifiers were removed during this process. As a further measure of quality control, two different people coded 10% of transcripts (i.e., double coding) to ensure consistency. Discrepancies found between any codes were resolved by the two coders and discussion with the primary investigator. Additionally, descriptive statistics pertaining to demographic and clinical information obtained during screening or interviews were computed, quality checked, and summarized.

### Study population

Interviews were conducted with a convenience sample of adult females with endometriosis who were recruited by qualitative research firms. A sample size needed for qualitative research is generally not defined [15] and instead strives to allow a large enough sample to allow the introduction of novel data, while still being small enough to permit deep analysis [16]. In order to achieve this balance in qualitative research, sample size determinations should be tailored to the particular study, and not necessarily based on numerical guidelines. Trained medical recruiters from firms located in Raleigh, North Carolina, and Dallas, Texas, recruited women in their databases to identify those who were eligible and interested in participating in the interviews. Interested women were evaluated using a recruitment screener consisting of 15 questions designed to ascertain eligibility and, if qualified, were scheduled for an interview.

Female participants from 18 to 49 years of age were eligible for inclusion based on the following criteria: were premenopausal or perimenopausal; reported having a menstrual cycle within the past 45 days/6 weeks of screening; could read and understand English; self-reported laparoscopic or other surgically confirmed endometriosis; and self-reported at least moderate pain associated with endometriosis. Exclusion criteria included the following: self-reported history of hysterectomy, oophorectomy, and/or current pregnancy; self-reported cessation of menstrual periods for 1 year or more owing to reasons other than pregnancy, breastfeeding, contraceptive use, or medical treatments for endometriosis; and being postmenopausal. Additional demographic data (e.g., age, race, education level) and clinical characteristics (e.g., time since endometriosis diagnosis, age at first period, endometriosis symptoms, and comorbidities currently experienced) were also captured during screening.

### Interview methods

Eleven interviews were conducted at each of the two sites (for a total of 22 interviews) in March 2019. Interviews followed a semistructured interview guide to ensure that data were collected in a systematic and consistent way to meet interview objectives, while also encouraging spontaneity of responses and fostering a conversational tone throughout the interviews. The interviews were designed to be concept elicitation interviews in which concepts relevant to participants were discussed (e.g., fatigue symptoms and impacts of fatigue). Concept saturation (i.e., point at which no new relevant concepts emerge) was also documented, consistent with the US Food and Drug Administration guidance on patient-reported outcomes [14].

Before the start of all interviews, participants underwent an informed consent discussion and provided written informed consent. Interviews began with a brief overview of the study and general questions intended to get participants talking about their endometriosis. The interview then progressed into discussion of key symptoms and the impact of endometriosis on participants’ lives, with a particular focus on endometriosis-related fatigue. Additionally, participants were asked to rate how bothersome their endometriosis-related fatigue was to them using a five-point scale ranging from “not at all bothersome” to “extremely bothersome.” To avoid biasing the participants or limiting their thinking, the interview included open-ended questions designed to ascertain ways in which participants described their endometriosis-related fatigue and its impact (i.e., day-to-day activities, social activities, physical activities, mood and emotions, relationships with family or partner, and work or school). Sample quotations of participants’ responses are provided for each of these respective areas in the Results section.

## Results

### Participant characteristics

A total of 22 women participated in the patient interviews. Participants had a mean age of 38.6 years (range, 27–48 years), with a wide range of age at diagnosis (15–37 years; mean: 24.7 years). The interview sample was racially and ethnically diverse, and all but one participant reported at least some college. Table 1 presents demographic and clinical characteristics of participants as reported at screening.

**Table 1:** Demographic and clinical characteristics collected at screening

Characteristic	Overall Sample (***N*** = 22)
Age (years)	
Mean (SD)	38.6 (6.5)
Range	27–48
Age at diagnosis (years)	
Mean (SD)	24.7 (6.2)
Range	15–37
Overall endometriosis-related pain in past 3 months^a^, n (%)	
Moderate	10 (45.5)
Severe	8 (36.4)
Extremely severe	4 (18.2)
Comorbidities, n (%)^b^	
Allergies	13 (59.1)
Asthma	6 (27.3)
Polycystic ovarian syndrome	5 (22.7)
Uterine fibroids	3 (13.6)
Irritable bowel disorder or irritable bowel syndrome	2 (9.1)
Adenomyosis	1 (4.5)
Current endometriosis symptoms, n (%)	
Pelvic/abdominal or lower back pain before or during periods that limits activities or requires medication	22 (100.0)
Backache, headache, joint pain, or other pain before or during periods	20 (90.9)
Heavy blood flow during periods	19 (86.4)
Fatigue, low energy	19 (86.4)
Gastrointestinal symptoms (e.g., diarrhea, constipation, bloating) before or during periods	19 (86.4)
Pelvic/abdominal or lower back pain between periods that limits activities or requires medication	16 (72.7)
Pain with sexual intercourse	15 (68.2)
Ethnicity, n (%)	
Hispanic	5 (22.7)
Race, n (%)	
Caucasian	10 (45.5)
African American	7 (31.8)
Other (not specified)	5 (22.7)
Education level, n (%)	
High school or equivalent	1 (4.5)
Some college but no degree	2 (9.1)
Associate’s degree/technical school	2 (9.1)
College degree	9 (40.9)
Some graduate school but no degree	5 (22.7)
Graduate or professional degree	3 (13.6)

*SD* Standard deviation

^a^Screener question: “Over the last 3 months, overall, how would you describe your endometriosis-related pain?”

^b^Total may exceed 100% because participants could report more than one comorbidity

At screening, participants reported many symptoms that they associated with endometriosis. Consistent with the screening criteria, all 22 participants reported “pelvic/abdominal or lower back pain” before or during periods and that this pain limited activities or required medication. Additionally, more than 80% of the sample reported experiencing “backache, headache, joint pain, or other pain” before or during periods; excessive/heavy menstrual bleeding, sometimes with clotting (menorrhagia); fatigue/low energy; and various gastrointestinal symptoms (e.g., diarrhea, constipation, bloating) before or during periods. Other symptoms reported by more than two-thirds of the sample at screening included nonmenstrual pelvic or lower back pain and dyspareunia. Among comorbidities queried, allergies were the most commonly reported (59.1%). Nearly 14% of the sample reported uterine fibroids. Six participants reported the use of some form of birth control, 3 participants reported the use of pain medication (1 specifically for heavy bleeding), and 1 participant reported the use of an antidepressant.

### Patient experiences with endometriosis

Interviews started with a brief discussion about participants’ experiences with endometriosis. Participants most commonly reported that initial endometriosis symptoms included pelvic/abdominal or lower back pain before or during periods (*n* = 18; 81.8%), heavy bleeding (*n* = 12; 54.5%), cramping (*n* = 9; 40.9%), and gastrointestinal symptoms (*n* = 5; 22.7%). Time since diagnosis ranged from 2 to 28 years. In general, participants reported experiencing symptoms well before receiving a formal diagnosis of endometriosis.

Participants reported a range of symptoms related to endometriosis, including fatigue (100%), pain with periods (86.4%), heavy bleeding (77.3%), nonmenstrual pelvic pain (50.0%), back pain and leg pain (40.9%), headaches/migraines (40.9%), cramping throughout the month (36.4%), gastrointestinal disturbances (constipation, diarrhea, pain with bowel movements) (36.4%), nausea (27.3%), bloating (22.7%), painful intercourse (18.2%), infertility (18.2%), and ovulation-related pain (9.1%). Nearly two-thirds of participants (*n* = 14; 63.6%) indicated that they were having periods monthly, and the remaining 8 participants reported having irregular/unpredictable periods. Among the 8 participants with irregular/unpredictable periods, 3 (13.6%) reported having periods fewer than 12 times a year, while 1 participant (0.5%) reported having periods more than once monthly; 4 participants (18.2%) said their periods were so unpredictable that they were unable to provide a response.

### Patient experiences with endometriosis-related fatigue

All 22 participants reported experiencing fatigue related to their endometriosis: 7 participants spontaneously reported fatigue as one of their current symptoms of endometriosis; the remaining 15 reported experiencing endometriosis-related fatigue upon probing. When describing their endometriosis-related fatigue, participants said that they felt “exhausted,” “drained,” “tired,” “lethargic,” “worn out,” and/or “weak.” Table 2 presents sample descriptions of participants’ endometriosis-related fatigue, which varied in the severity and frequency. Most participants reported their worst severity of fatigue before or during the first few days of their period (*n* = 19). Other participants reported experiencing their worst fatigue throughout the month (*n* = 2) or during ovulation (*n* = 1).

**Table 2:** Participant Descriptions and Reported Bothersomeness of Endometriosis-Related Fatigue

**Quotation**	
**Participant descriptions**	
“Like somebody completely drained your energy out of you. And it’s really hard to like function and complete your daily tasks as you normally would if it wasn’t that time of the month.”	
“The best way I guess I could describe it is, I guess it’s, when you’re pregnant and you’re sleepy and you’re tired. It just comes on like that.”	
“Lethargic, more just tired, I mean just feeling drained. No mental clarity.”	
“You just feel really sleepy and tired and run-down.”	
It’s mental fatigue, it’s physical exhaustion, and especially the first day or two I come home, I need to take a nap. I’m just spent. And so it seems like I can’t get enough sleep.”	
**Participant-reported bothersomeness**	
“Extremely bothersome. It’s curtailed my work, my social life, me wanting to get up and just do everyday things. It’s impacted my life in so many ways. The pain I can deal with, but the fatigue is what really doesn’t allow me to do the laundry, clean up, go run these errands, get out of bed, take a shower.”	
“Quite bothersome … But as you talk through like all the things that it affects, it’s like ‘This really sucks.’ I try not to think about it because you don’t want to get in a negative but it literally affects every part of my life in one way or another.”	
“I would say quite bothersome. If it’s something that stops you from doing what you’re doing. If it interrupts your daily activities, your daily routine, your plans, that’s quite bothersome.”	
“Extremely bothersome. Just not being able to do anything. I’m a go, go, go type person, and me having to shut my whole world down, it’s hard for me. And I’m thinking maybe it’s a way of saying I need to sit down and rest, but it bothers me a lot.”	

Qualitative descriptions of bother associated with fatigue are provided in Table 2. Most participants considered their endometriosis-related fatigue to be “quite bothersome” (*n* = 12) or “extremely bothersome” (*n* = 4). The remaining participants noted that it was “somewhat bothersome” (*n* = 5) or only “a little bothersome” (*n* = 1). No participant stated that it was “not at all bothersome.” All participants were asked how endometriosis-related fatigue impacted their lives. The most commonly reported impacts were on day-to-day activities, social activities, physical activities, mood and emotions, relationships with family or partner, and work or school (Table 3). No new impacts of endometriosis-related fatigue were identified in the second set of interviews (Dallas), a finding that suggests that concept saturation was achieved [17]. Each of these impacts are further explored in the subsequent sections with sample quotations given in tables to provide perspective directly from the participants.

**Table 3:** Frequency (%) of Endometriosis-Related Fatigue Impacts

Impact, n (%)	Raleigh (***n*** = 11)	Dallas (***n*** = 11)	Total (***N*** = 22)
Day-to-day activities	11 (100.0)	11 (100.0)	22 (100.0)
Social activities	10 (90.9)	11 (100.0)	21 (95.5)
Physical activities	10 (90.9)	9 (81.8)	19 (86.4)
Mood and emotions	10 (90.9)	11 (100.0)	21 (95.5)
Relationships with family or partner	10 (90.9)	11 (100.0)	21 (95.5)
Work or school	9 (81.8)	11 (100.0)	20 (90.9)

#### Day-to-day activities

All 22 participants reported that endometriosis-related fatigue negatively impacted their day-to-day activities, such as delaying chores (e.g., laundry, grocery shopping, cleaning, cooking) and self-care activities (e.g., brushing teeth, bathing, washing hair), as shown by the sample quotations in Table 4.

**Table 4:** Impacts of Endometriosis-Related Fatigue on Day-to-Day, Social, and Physical Activities

**Quotation**	
**Day-to-day activities**	
“It makes it difficult to do normal things. Just taking care of my kids, getting them to school, making lunches, cleaning up, doing just normal everyday activities.”	
“So simply like going to the grocery store, I’ve skipped out on stuff like that. And like, ‘Oh, I’ll wait till tomorrow,’ just because of how bad I’m feeling. It can be simple things.”	
“I go to bed with the kitchen a mess because I’m tired and normally I don’t do that. I like to wake up with a kitchen clean.”	
“Well if I needed to do laundry on that day, during that period, it probably wouldn’t get done.”	
“I won’t even be able to take a shower and brush my teeth.”	
“And I have to kind of like force myself and really psych myself up to, ‘Hey, the dishes ought to be done. Hey, the laundry has to be folded.’ And so that’s a battle too.”	
**Social activities**	
“I’d rather stay at home than to go and be social.”	
“Every once in a while, if I have girlfriends that want to get together and socializing in the evenings, I’ll skip on that to go to bed because I’m tired.”	
“I don’t schedule anything [during my period]. I try to pinpoint my period. If I know it’s coming close, I don’t schedule anything.”	
“If I have something going on that can be canceled, you better believe, it gets canceled.”	
“I don’t do anything. I will sit at home. I don’t really even go to church with this. I don’t feel like doing nothing.”	
“I’ve spent so many years of my life trying to plan anniversary trips or family vacations around my period because I’m so miserable.”	
“I just don’t want to be around people because I don’t want to go off the crazy end, so I try to avoid social encounters.”	
**Physical activities**	
“I don’t have energy to work out. I try to work out 2 to 3 days a week, and I don’t have energy for that. I don’t do anything physical.”	
“I’m not as motivated to go to the gym to work out.”	
“I just feel so bad today that I’m not going to work out. Like I’ve gone a week without working out because my cycle’s about to come and I’m just feeling really bad.”	
“I have a dog. Sometimes I don’t want to go for a long walk because I just can’t do it today.”	
“Well, I do not want to run. I typically run after my shift with my partner for a little while before I go home. And on days where I’m exhausted from my period, I’m like I tap out.”	
“It keeps me on the sofa instead of [being active] … because really I’m an active person. I like hiking and cycling, and I don’t get many opportunities to get out because of my work schedule, and so when I miss that opportunity because I’m just feeling tired and bummed, that’s what it mostly impacts.”	

#### Social activities

Nearly all participants (*n* = 21; 95.5%) reported canceling or modifying social plans because of endometriosis-related fatigue. Additionally, some participants reported trying to “plan for” their periods in order to schedule trips and make social plans around these dates, whereas other participants reported withdrawing from others socially because of their endometriosis-related fatigue. Table 4 provides example quotations on impacts of endometriosis-related fatigue on social activities.

#### Physical activities

Most participants (*n* = 19; 86.4%) reported a negative impact on their physical activities because of endometriosis-related fatigue. Participants reported staying indoors and avoiding physical activities, such as going to the gym, jogging, or walking up the stairs, when experiencing endometriosis-related fatigue. Table 4 provides sample quotations on impacts of endometriosis-related fatigue on physical activities.

#### Mood and emotions

Nearly all participants (*n* = 21; 95.5%) reported a negative impact on their mood and emotions because of endometriosis-related fatigue. The most commonly reported impact of fatigue was feeling irritable or moody. Some participants reported also feeling depressed because of their fatigue, as seen by the sample quotations presented in Table 5.

**Table 5:** Impacts of Endometriosis-Related Fatigue on Mood and Emotions, Relationships With Family/Partner, and Work/School

**Quotation**	
**Mood and emotions**	
“I would say I’m a little more depressed during my period. A little bit more emotional. More sensitive.”	
“I think fatigue, typically when I’m tired, I’m grouchy.”	
“It almost makes me feel like something’s a big deal when it’s not. You can’t just like let something go. It irritates you.”	
“[When fatigued, I am] Short tempered, irritable, less understanding and patient.”	
“It’s more just kind of feeling disappointed because you can’t do as much as you want to do.”	
**Relationships with family or partner**	
“Well I decide not to take my children to play with friends or that type of a thing.”	
“Sometimes I don’t want to get up and do things with my kids or take them places.”	
“And as far as my husband, I probably just am not around him as much. I just go to my room and watch TV in my bed.”	
“When I am at home, I’m not as interactive and playful with my kids. My husband takes more of the responsibility instead of me helping out as much as I would be.”	
“I feel like I’m failing my kids partly like at school just because I’m like, ‘You have this due.’ And it’s one of those reminders that like I can tell myself 12 times, write it down on the calendar, and I just feel like I’m so tired that I’m forgetting until it like clicks again, oh that’s right.”	
“Our sex life sometimes sucks. Because I’m just like, ‘I don’t feel like it.’”	
**Work or school**	
“There are days where I modify my activities, meaning I may have had plans to go install cabinets [for work] on a certain day and I might end up staying back and working in the office instead.”	
“Like mental capacity. Like you just can’t, you’re not focusing as well. I’m not focusing as well because I can tell that I’m just tired. So … my mentality for that day will be like, ‘I just need to get home. I just need to get through this day.’ So productivity goes down, overall attitude is down, and then level of focus.”	
“On those bad days when I’m at work, it’s hard to focus during those days, and so I would say sometimes I do leave the office early because I’m useless.”	
“There have been days where I’ll stay home. I don’t go to work.”	

#### Relationships with family or partner

Most of the participants (n = 21; 95.5%) reported that endometriosis-related fatigue impacted their relationships with family and partners in a variety of ways, as shown by the sample quotations in Table 5. Participants reported limiting the amount of time spent together with their family and decreasing intimacy with their partner, Additionally, participants limited social interaction with their children.

#### Work or school

Most participants (*n* = 20; 90.9%) reported a decrease in their ability to focus, productivity, and quality of work because of fatigue. Additionally, some participants reported skipping work or leaving early or modifying their tasks owing to endometriosis-related fatigue. Table 5 provides sample quotations on impacts of endometriosis-related fatigue on work or school.

## Discussion

In this study, all 22 participants reported experiencing endometriosis-related fatigue and provided new insight on how this symptom impacted their lives. The varied terms used by the participants to describe this concept, including feeling “exhausted,” “drained,” “tired,” “lethargic,” “worn out,” and “weak,” underscore the burden of this symptom from the patient’s perspective. In addition to the various labels for fatigue, participants also differed in the reported severity of their endometriosis-related fatigue. Most participants reported that their fatigue was most severe before or during the first few days of their period; only three participants reported experiencing their most severe fatigue at other times of the month, including during ovulation. Notably, participants reported that decreases in pain and heavy bleeding were associated with decreased fatigue. This relationship between pain and fatigue suggests that fatigue could be treated through the management of pain and, to some extent, heavy bleeding. The most commonly reported impacts of endometriosis-related fatigue were on day-to-day activities (e.g., household chores and self-care activities); social activities; mood or emotions (e.g., feeling irritable or withdrawn); relationships with family and partner (e.g., time spent with family, intimacy with partner); work or school (e.g., ability to focus, the need to skip work or leave early); and physical activities (e.g., exercise). These data indicate that endometriosis-related fatigue has a pervasive impact on the functioning of women living with this condition.

The results of the patient interviews presented here provide descriptive detail about the impact of endometriosis-related fatigue that has been largely absent from the literature. Although descriptive characterizations may be scarce, the prevalence of endometriosis-related fatigue has been documented by recent studies with large patient populations [6–9]. For example, Surrey et al. [9] analyzed the effect of a nonpeptide gonadotropin-releasing hormone (GnRH) antagonist on the reduction of fatigue in a double-blind, multicenter, randomized controlled trial (RCT) that enrolled 860 women with moderate or severe endometriosis-related pain. Nearly three-quarters (74%) of the participants in this RCT reported a moderate or severe level of fatigue at baseline. Greater than half of the RCT participants observed difficulty initiating activities and with physical functioning due to their fatigue, which was consistent with our finding that most patients avoided engaging in physical activity. The authors noted an increase in fatigue in the presence of nonmenstrual pelvic pain, dysmenorrhea, or dyspareunia and observed that treating these major symptoms of endometriosis may significantly reduce fatigue. Ramin-Wright et al. [6] explored the frequency of fatigue in a matched case-control study consisting of 560 women with confirmed endometriosis and 560 women without endometriosis as the control. Women with endometriosis in this case-control study reported greater than double the prevalence of frequent fatigue as the control group (50.7% vs. 22.4%; *P* < 0.001), and this difference remained significant even after controlling for confounding effects. In a panel-based survey of 1269 women with endometriosis conducted by Soliman et al. [8], 75% of the participants reported that they had experienced fatigue/weariness/anemia. More than 60% of participants in the survey included this concept among the four symptoms they experienced most frequently, along with the hallmark symptoms of pain/cramping during menstrual period, anxiety/stress, and lower back pain. Ultimately, more than 40% of the survey sample indicated that the fatigue/weariness/anemia they experienced was extremely bothersome to them, and the concept was also associated with significant worsening in emotional well-being and social support. The worsening of emotional well-being was consistent with our results, where nearly all participants reported feeling irritable, moody, or depressed because of their endometriosis-related fatigue. In a study involving 1361 women (610 diagnosed with endometriosis and 751 without endometriosis) recruited via the Danish Endometriosis Association, advertisements, social media, and a hospital clinic, 54.8% of the participants with endometriosis identified “feeling tired/lack of energy” as a disturbing symptom at work compared with 23.9% of the women without endometriosis [7].

### Limitations

Data for this study came from interviews exploring past and ongoing experiences with endometriosis. Such qualitative data are subjective by nature but do provide a richness to the understanding of patients’ experiences with endometriosis. All clinical information, including diagnosis of endometriosis, was self-reported and was not confirmed in the participants’ medical records. However, participants were required to confirm during screening that diagnosis was based on surgical confirmation. Additionally, the small sample size, the similar geographic location of both study sites (southern United States), and the restriction of eligibility to only women with moderate to severe endometriosis-related pain may limit the generalizability of findings to a broader population or different cultures. Concept saturation was achieved, which suggests the sample size was adequate for characterizing the qualitative impacts of endometriosis-related fatigue [18].

## Conclusion

Endometriosis-related fatigue was reported by 100% of the sample of women with endometriosis and moderate to severe levels of pain. Endometriosis-related fatigue negatively impacted multiple areas of many women’s lives, particularly day-to-day activities, mood and emotions, social activities, relationships with family or partner, work or school, and physical activities. These data, coupled with results of previous work, highlight the significance and burden of this symptom in women with endometriosis and suggest future studies should measure any changes in fatigue that may be associated with treatment for endometriosis.

## Data Availability

The datasets used and/or analyzed during the current study are available from the corresponding author on reasonable request.

## Abbreviations

GnRH: Gonadotropin-releasing hormone
IDI: In-depth interview number
SD: Standard deviation
US: United States
